# Supplementary material for: BNIP3L-Dependent Mitophagy Promotes HBx-Induced Cancer Stemness of Hepatocellular Carcinoma Cells via Glycolysis Metabolism Reprogramming
Source: Cancers (Basel). 2020 Mar 11;12(3):655. doi: 10.3390/cancers12030655 (PMC7139741; doi:10.3390/cancers12030655)
Supplement: Supplementary file 1 [file cancers-12-00655-s001.pdf]

cancers

Supplementary Materials:

BNIP3L-Dependent Mitophagy Promotes HBx-Induced Cancer Stemness of Hepatocellular Carcinoma Cells via Glycolysis Metabolism Reprogramming

Yuan-Yuan Chen [1,2,†], Wei-Hua Wang [1,†], Lin Che [1,†], You Lan [1], Li-Yin Zhang [1], Deng-Lin Zhan [1], Zi-Yan Huang [1], Zhong-Ning Lin [1,*] and Yu-Chun Lin [1,*]

Table 1. Primers used for qRT-PCR in this study.

Gene	Primers
ABCG2	FP: 5'-ATA TTA TCG AAT ATC AAT GGG ATC A-3'<br>RP: 5'-CAA TGA AAA TCT TCA GGA GAT A- 3'
OCT4	FP: 5'- CAC CAG GGC GTG ATG GT -3'<br>RP: 5'-ACC ACA CTC GGA CCA CAT C-3'
NANOG	FP: 5'-AAC CTC AGC TAC AAA CAG GT-3'<br>RP: 5'-AGG TCT GGT TGC TCC ACA T-3′
KLF4	FP: 5'-CTC CAT TAC CAA GAG CTC AT-3'<br>RP: 5'-GGT AAG GTT TCT CAC CTG T-3'
BMI1	FP: 5'-ACA TTC CTT CTG TAA AAC GTG-3'<br>RP: 5'-CAT TGG CAG CAT CAG CAG-3'
GLUT1	FP: 5'-GTG GGC ATG TGC TTC CAG TAT-3′<br>RP: 5'-CAG CTC CTC GGG TGT CTT GT-3′
HK2	FP: 5'-TTC TTG GCC TTG GAC CTT G-3′<br>RP: 5'-CCA GAT GCC TTG AAG CCT TTT-3′
PFKL	FP: 5'-GCT GGG CGG CAC TAT CAT T-3'<br>RP: 5'-TCA GGT GCG AGT AGG TCC G-3'
LDHA	FP: 5'-CCA AGC TGG TCA TTA TCA CGG-3'<br>RP: 5'-CAT TCC ACT CCA TAC AGG CAC-3′
ACTB	FP: 5′-CAC CAG GGC GTG ATG GT-3′<br>RP: 5′-CTC AAA CAT GAT CTG GGT CAT-3′

FP: forward primer; RP: reverse primer.

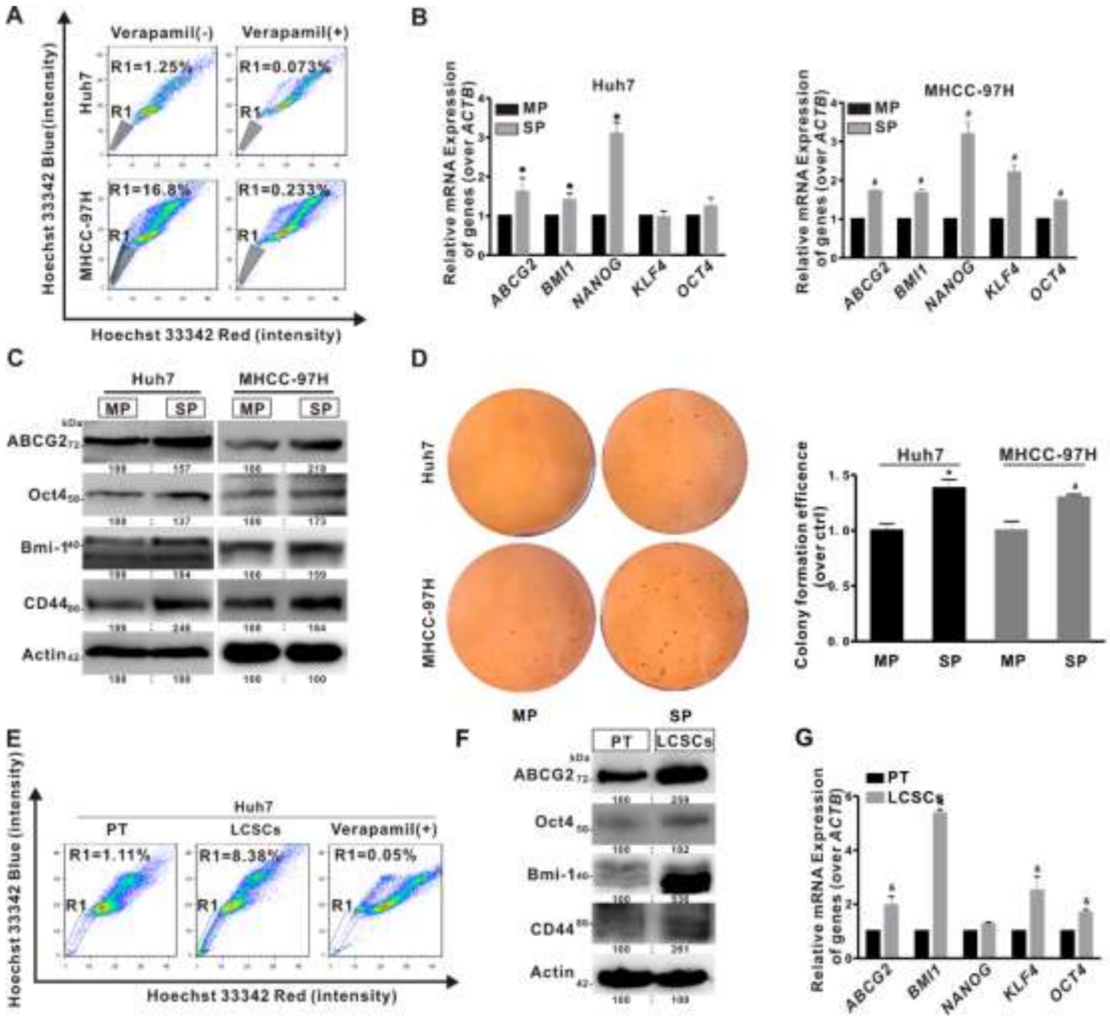

Figure. S1 Liver cancer stemness characteristics of HCC cells. (**A**) Percentage of the sorted SP cells (R1 gate) in Huh7 and MHCC-97H cells was detected by Flows cytometry (FCM). Representative images of FCM were shown. R1 gate represented SP cells and others were MP cells. (**B**) The mRNA levels of cancer stemness-related genes in SP and MP cells in Huh7 cells (Left) and MHCC-97H cells (Right). (**C**) The expression levels of cancer stemness-related proteins in SP and MP cells in Huh7 cells (Left) and MHCC-97H cells (Right). (**D**) The self-renewal capacity of SP and MP cells in two HCC cell lines was measured by colony formation assay. Representative images of colonies (Left) were shown in Huh7 cells (Upper) and MHCC-97H cells (Lower), while the quantified data were shown in bar graph (Right). (**E**–**G**) LCSCs were enriched in Huh7 cells by sphere-formation assay, and normally cultured Huh7 cells served as the parental cells (PT) control group. Percentage of the sorted SP cells (R1 gate) (**E**), the expression levels of cancer stemness-related proteins (**F**), and the level of cancer stemness-related genes (**G**) in PT and LCSCs of Huh7 cells were shown. The gray value of band was assessed by image-pro plus 6.0. The relative expression levels of indicated proteins were shown. The target gene expression was normalized to *ACTB*. * $P < 0.05$ as compared with MP or PT group. SP: side population, MP: main population.

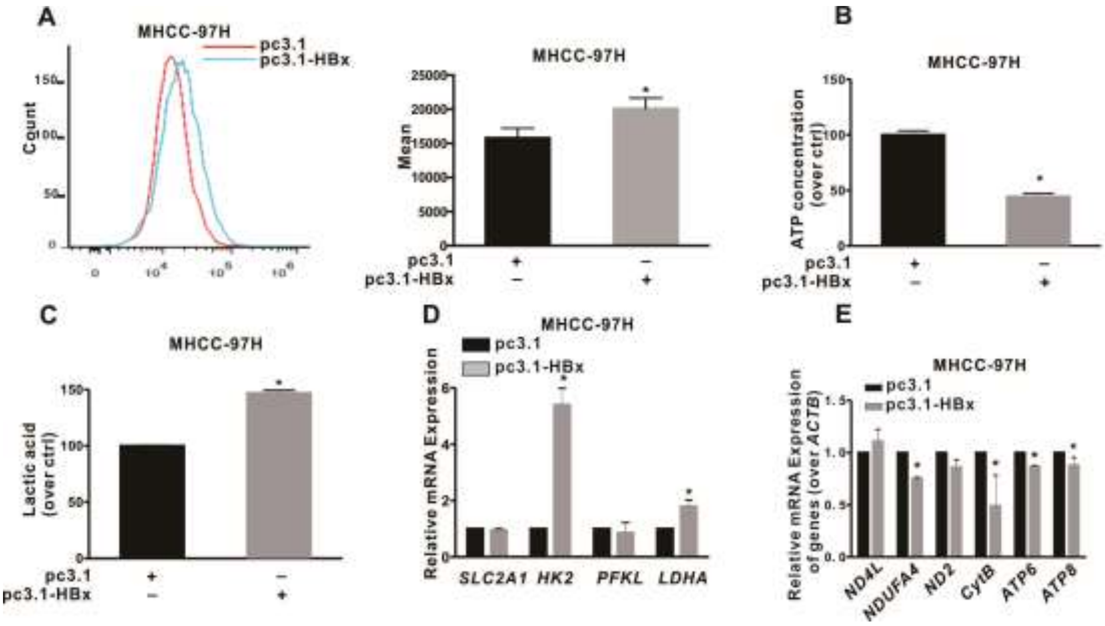

Figure S2. Glycolysis metabolic reprogramming was induced in HBx-expressing MHCC-97H cells. MHCC-97H cells without or with HBx-expressing were transiently transfected with pcDNA3.1 or pcDNA3.1-HBx (1 μg/mL) for 8 h, and followed by restored culture for another 48 h. (**A**) Glucose transport activity was evaluated by FCM (Left). The mean data was shown in bar graph (Right). The levels of the intracellular ATP content (**B**), the extracellular lactate secretion (**C**), the mRNA levels of glycolysis-related genes (**D**), and the mRNA levels of OXPHOS-related genes (**E**) were detected in MHCC-97H cells without or with HBx-expressing. The target gene transcription was normalized to *ACTB*. * $P < 0.05$ as compared with pc3.1 group. pc3.1: pcDNA3.1 transfection without HBx-expressing. pc3.1-HBx: pcDNA3.1-HBx transfection with HBx-expressing.

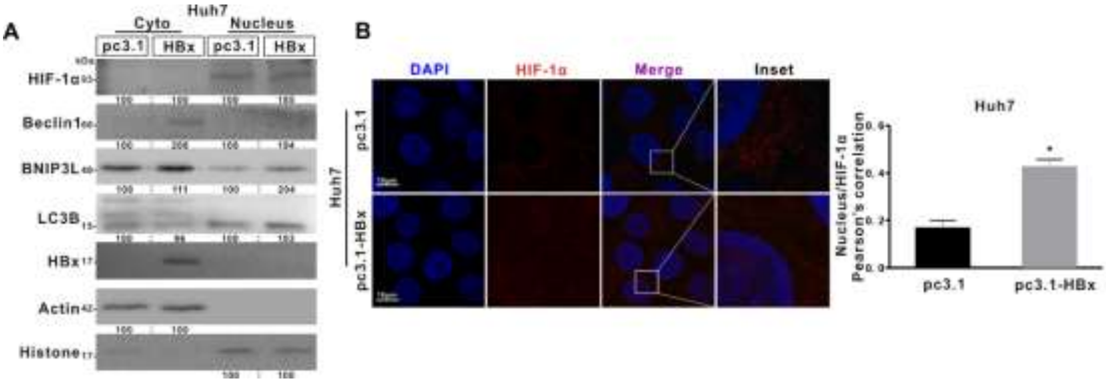

Figure S3. HIF-1α upregulation and nuclear translocation in HBx-expressing Huh7 cells. Huh7 cells without or with HBx-expressing were transiently transfected with pcDNA3.1 or pcDNA3.1-HBx (1 μg/mL) for 8 h, and followed by restored culture for another 48 h. (**A**) The expression of BNIP3L-dependent mitophagy-related proteins in the cytoplasmic (Cyto) and nuclear fractions. The gray value of band was assessed by image-pro plus 6.0. The relative expression levels of indicated proteins were shown. (**B**) Representative images of the immunofluorescence co-staining for DAPI (blue) and HIF-1α (red) (Left). Pearson's correlation for the co-localization of nucleus with HIF-1α was shown in bar graph (Right). Scale bar represents 10 μm. * $P < 0.05$ as compared with pc3.1. pc3.1: pcDNA3.1 transfection without HBx-expressing. pc3.1-HBx: pcDNA3.1-HBx transfection with HBx-expressing.

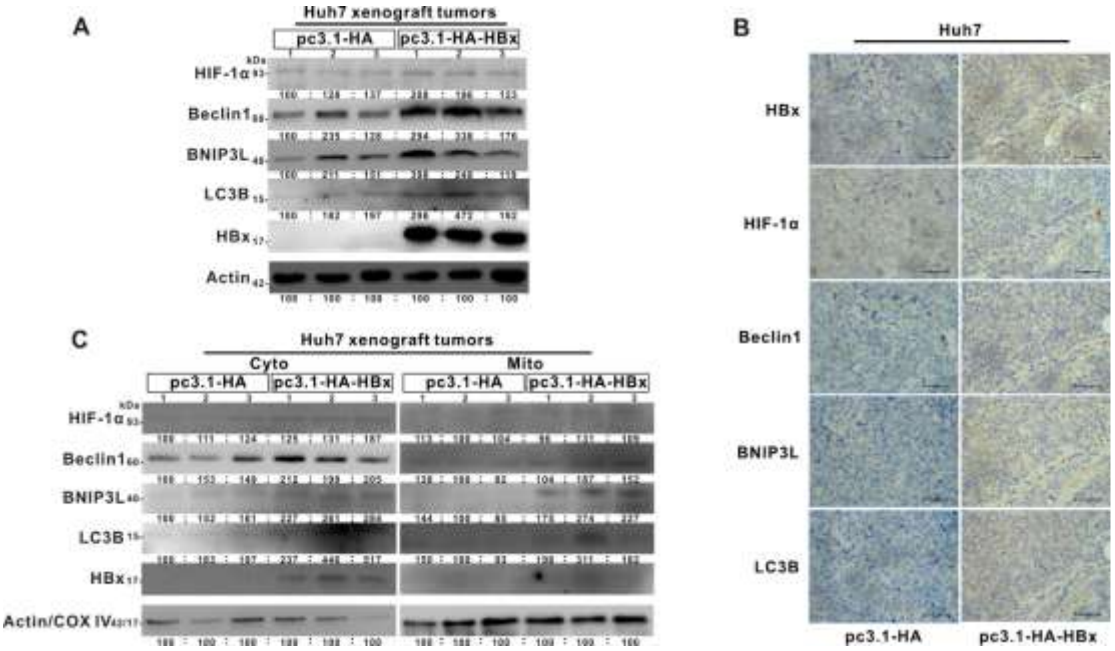

Figure S4. BNIP3L-dependent mitophagy was induced in HBx-expressing Huh7 xenograft tumors. The HCC xenograft tumors with or without HBx-expressing were formed by Huh7-/MHCC-97H-pcDNA3.1-HA-HBx or Huh7-/MHCC-97H-pcDNA3.1-HA cells in BALB/c nude mice for 24 days (n = 6). (**A**) The expression levels of the BNIP3L-dependent mitophagy-related proteins in the Huh7 xenograft tumors with or without HBx-expressing. (**B**) Immunohistochemical staining of the BNIP3L-dependent mitophagy-related proteins in the Huh7 xenograft tumors with or without HBx-expressing. Scale bar represents 50 μm. (**C**) The protein expression of BNIP3L-dependent mitophagy in cytoplasmic (Cyto) and mitochondrial (Mito) fractions of Huh7 xenograft tumors with or without HBx-expressing. The gray value of band was assessed by image-pro plus 6.0. The relative expression levels of indicated proteins were shown. pc3.1-HA: pcDNA3.1-HA transfection without HBx-expressing. pc3.1-HA-HBx: pcDNA3.1-HA-HBx transfection with HBx-expressing.

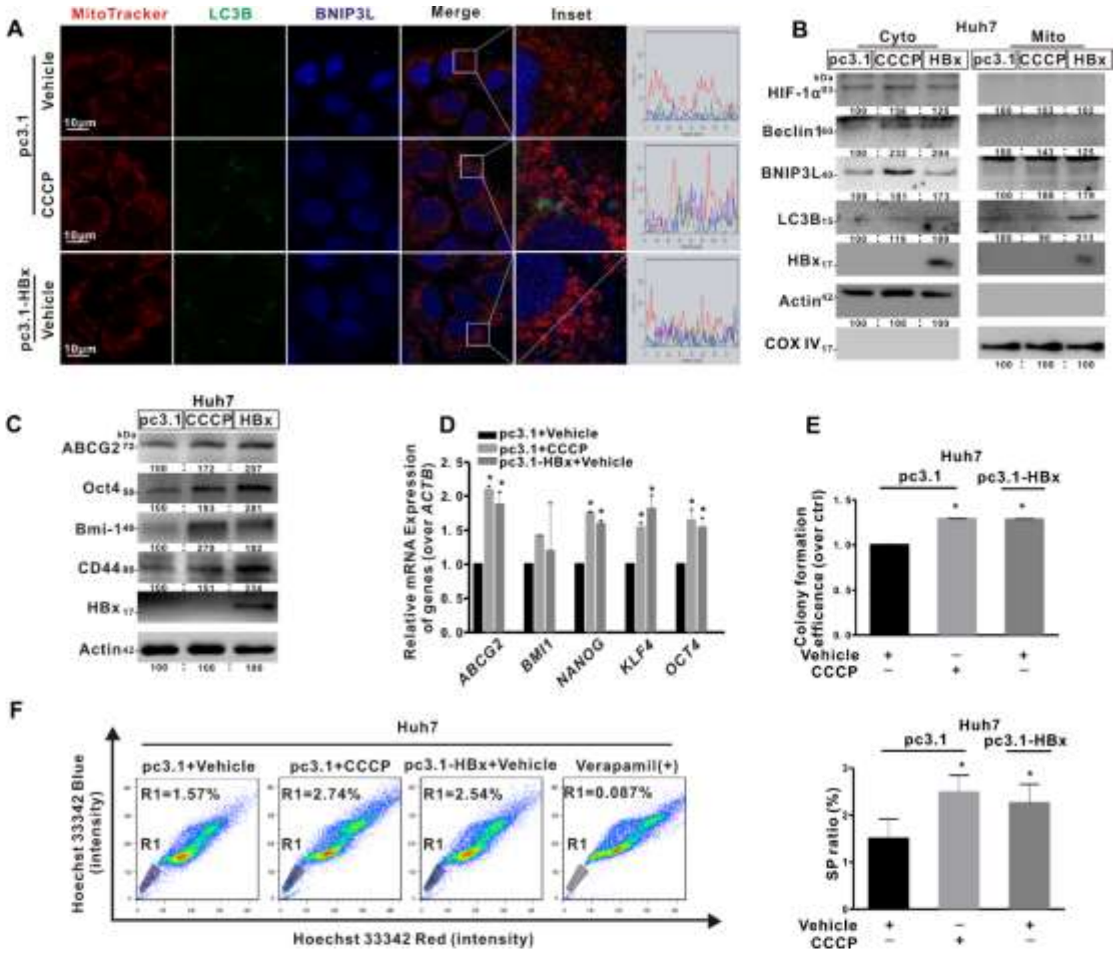

Figure S5. BNIP3L-dependent mitophagy was involved in maintaining the liver cancer stemness. Huh7 cells without or with HBx-expressing were transiently transfected with pcDNA3.1 or pcDNA3.1-HBx (1 μg/mL) for 8 h, pretreated with CCCP at 20 μM for 3 h or not. (**A**) Representative images of the immunofluorescence co-staining for MitoTracker (red), BNIP3L (blue), and LC3B (green). The profiles of representative lines trace the intensities of fluorescence signals. Fluorescence curves with line intensity profile generated by Zen 2012 software were shown. Scale bar represents 10 μm. (**B**) The BNIP3L-dependent mitophagy-related proteins in cytoplasmic (Cyto) and mitochondrial (Mito) fractions. (**C**) The expression levels of cancer stemness-related proteins. The gray value of band was assessed by image-pro plus 6.0. The relative expression levels of indicated proteins were shown. (**D**) The mRNA levels of cancer stemness-related genes. The target gene transcription was normalized to *ACTB*. (**E**) The self-renewal capacity was measured by colony formation assay. (**F**) Percentage of the sorted SP cells (R1 gate) were detected by FCM (Left). Quantitative results were shown in bar graph (Right). SP: side population. R1 gate represented SP cells. * $P < 0.05$ as compared with pc3.1+Vehicle group. pc3.1: pcDNA3.1 transfection without HBx-expressing. pc3.1-HBx: pcDNA3.1-HBx transfection with HBx-expressing.

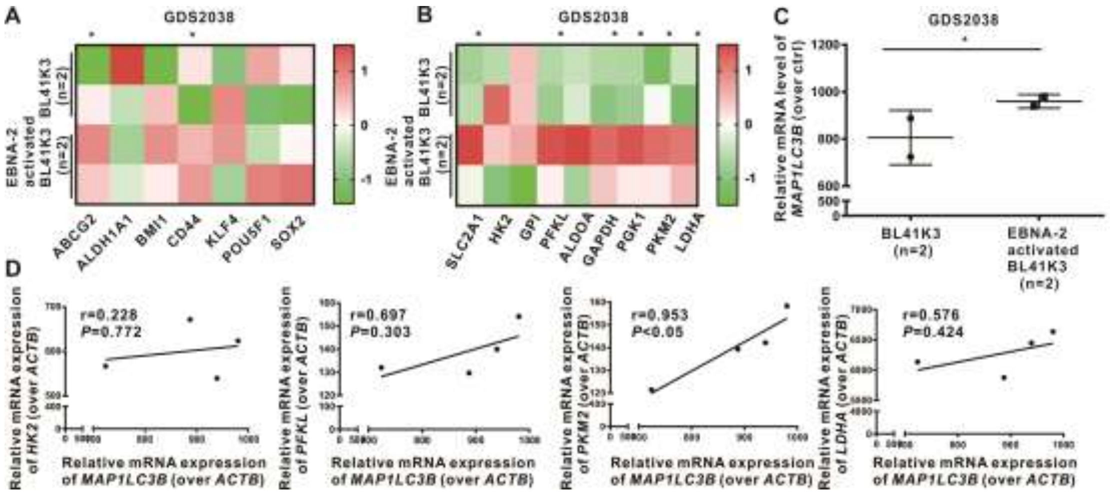

Figure S6. GEO analysis of cancer stemness, glycolysis, and mitophagy related genes in EBNA-2 overexpressing BL41K3 cells. The relative mRNA levels of indicated genes were obtained from NCBI, GEO database (GSD2038). The samples were derived from Epstein-Barr virus (EBV) negative B-cell lines BL41K3 (n = 2) and EBV nuclear antigen 2 (EBNA-2) overexpressing BL41K3 cells (n = 2). The heat-maps of the relative mRNA levels of cancer stemness-related genes (**A**) and glycolysis-related metabolism genes (**B**), and the relative mRNA levels of *MAP1LC3B* gene (**C**) were shown. (**D**) The linear correlation of *MAP1LC3B* with glycolysis-related metabolism genes were shown. * $P < 0.05$ as compared with BL41K3 cells.

Figure S7. The original full scan of each WB (as shown below, right panels)and the cropped area had been indicated (in red rectangle). Western blot bands were visualized using the Azure Biosystems.

Fig 1

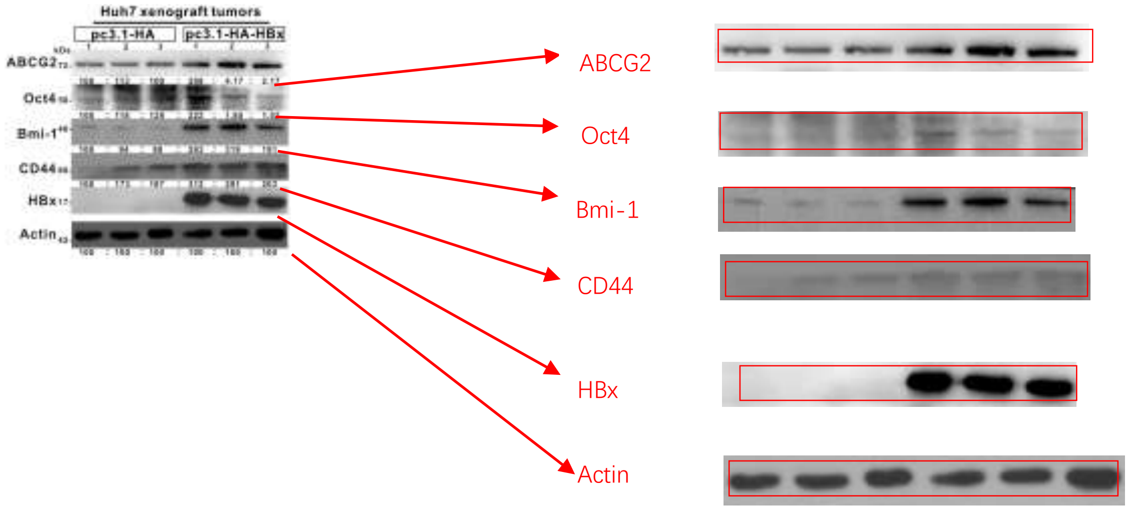

Fig.2

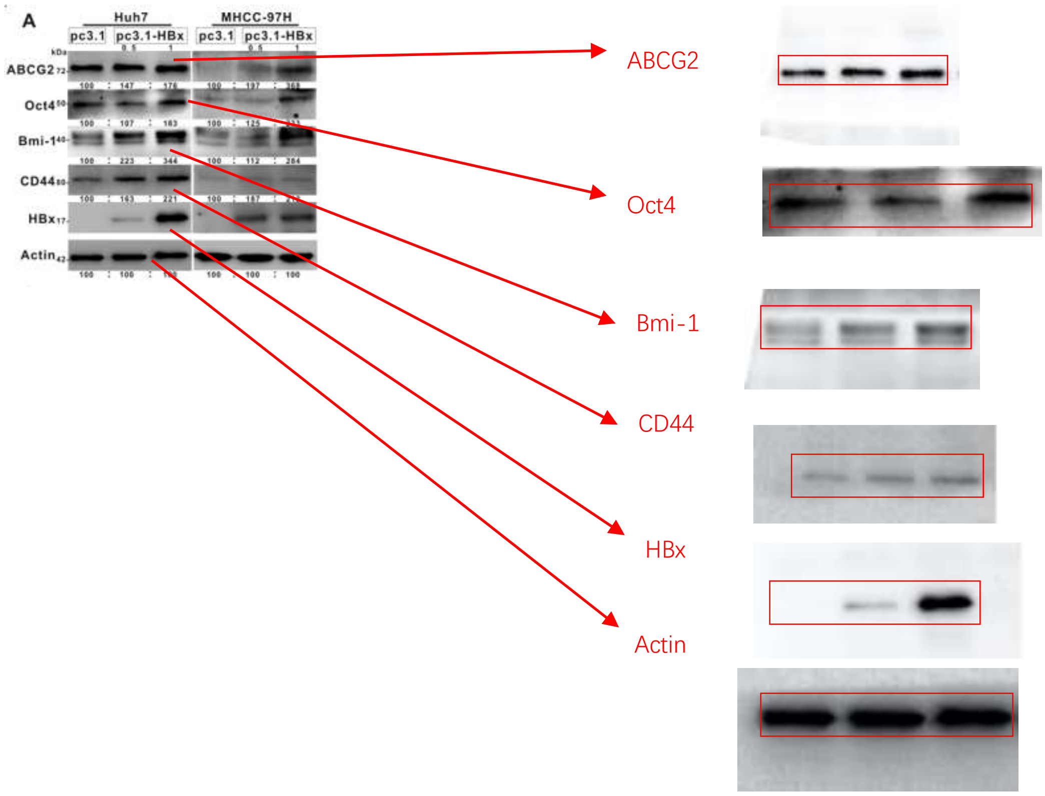

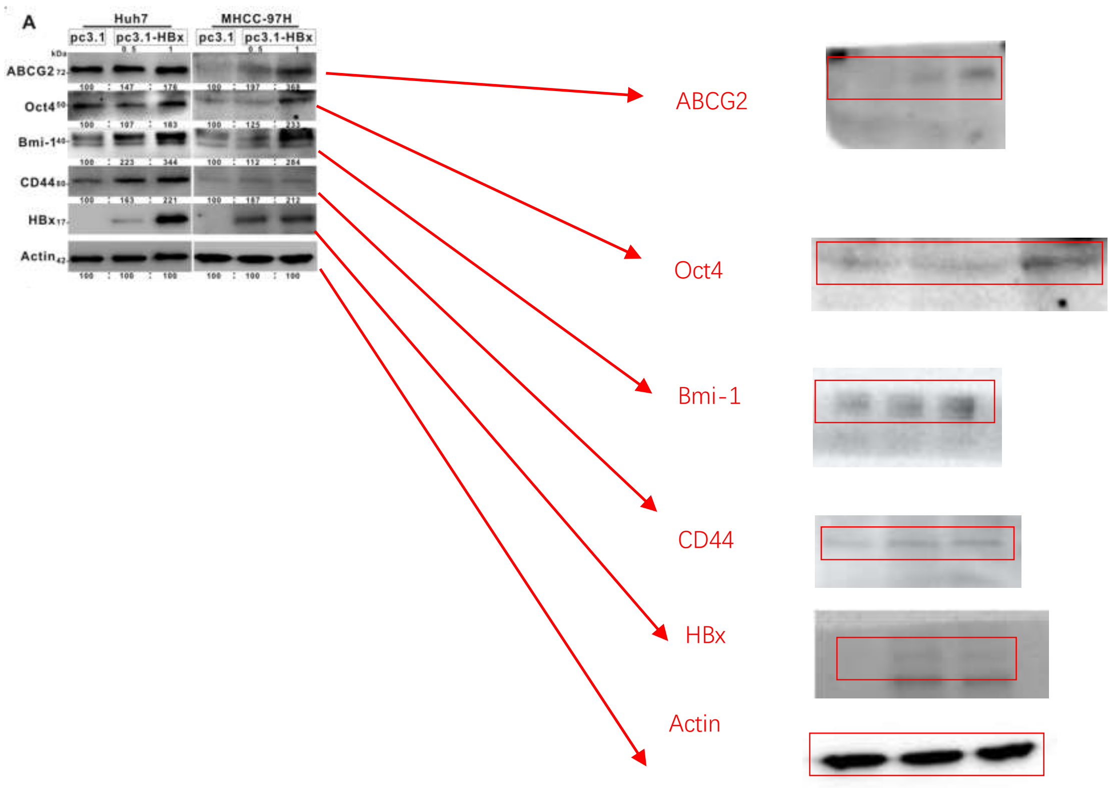
A
Huh7
MHCC-97H
pc3.1
pc3.1-HBx
pc3.1
pc3.1-HBx
ABCG2
Oct4
Bmi-1
CD44
HBx
Actin
ABCG2
Oct4
Bmi-1
CD44
HBx
Actin

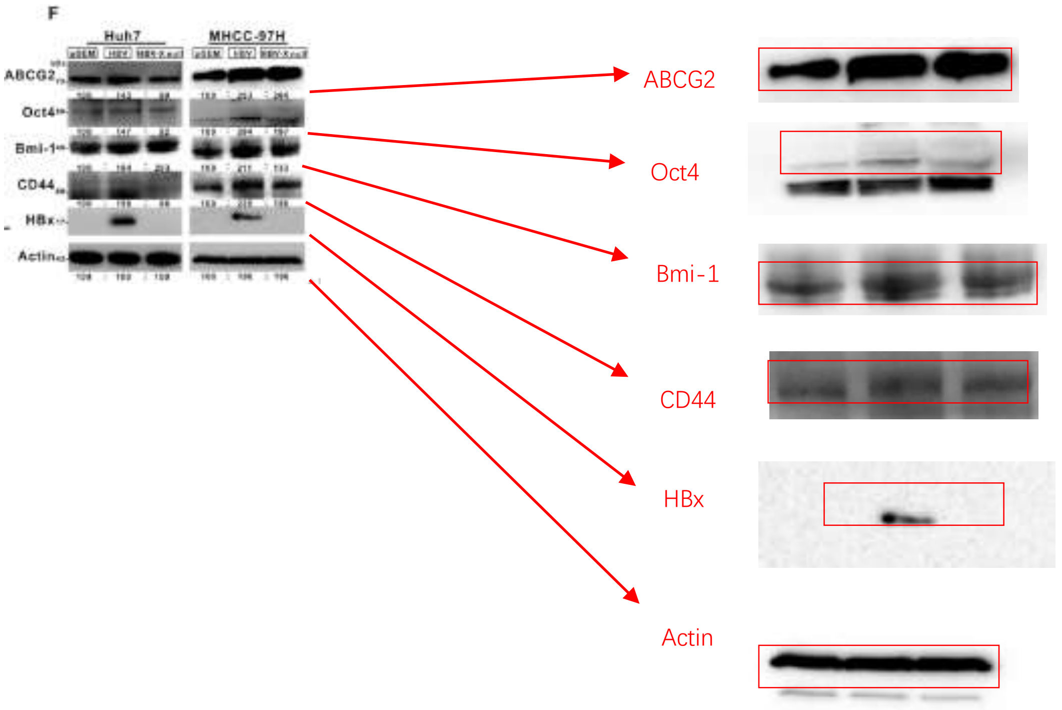
F
Huh7
MHCC-97H
ABCG2
Oct4
Bmi-1
CD44
HBx
Actin
ABCG2
Oct4
Bmi-1
CD44
HBx
Actin

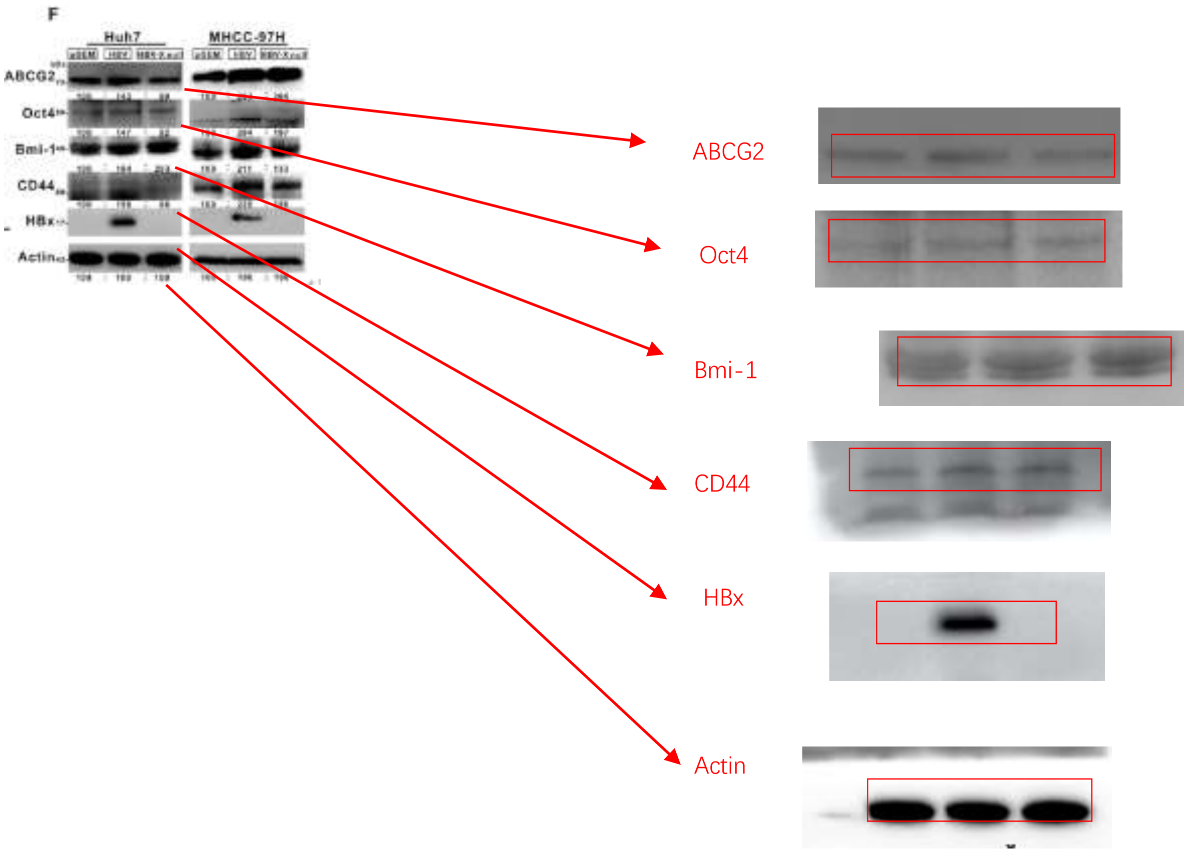
F
Huh7
MHCC-97H
ABCG2
Oct4
Bmi-1
CD44
HBx
Actin
ABCG2
Oct4
Bmi-1
CD44
HBx
Actin

Fig 4

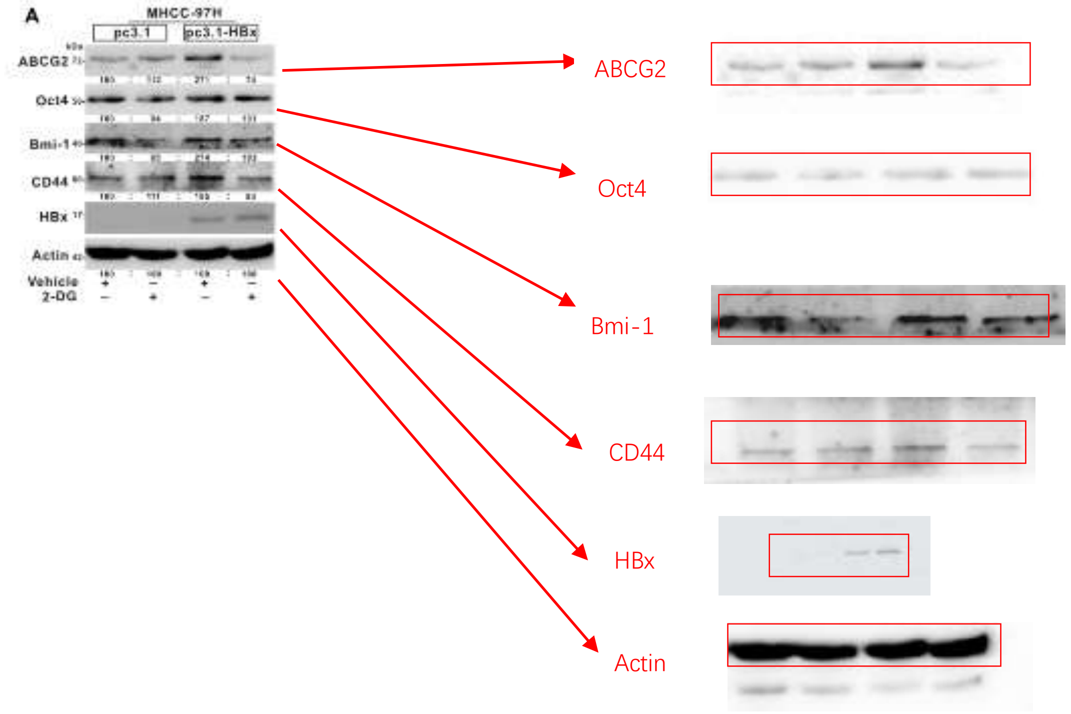
A
MHCC-97H
pc3.1
pc3.1-HBx
ABCG2
Oct4
Bmi-1
CD44
HBx
Actin
Vehicle
2-DG
ABCG2
Oct4
Bmi-1
CD44
HBx
Actin

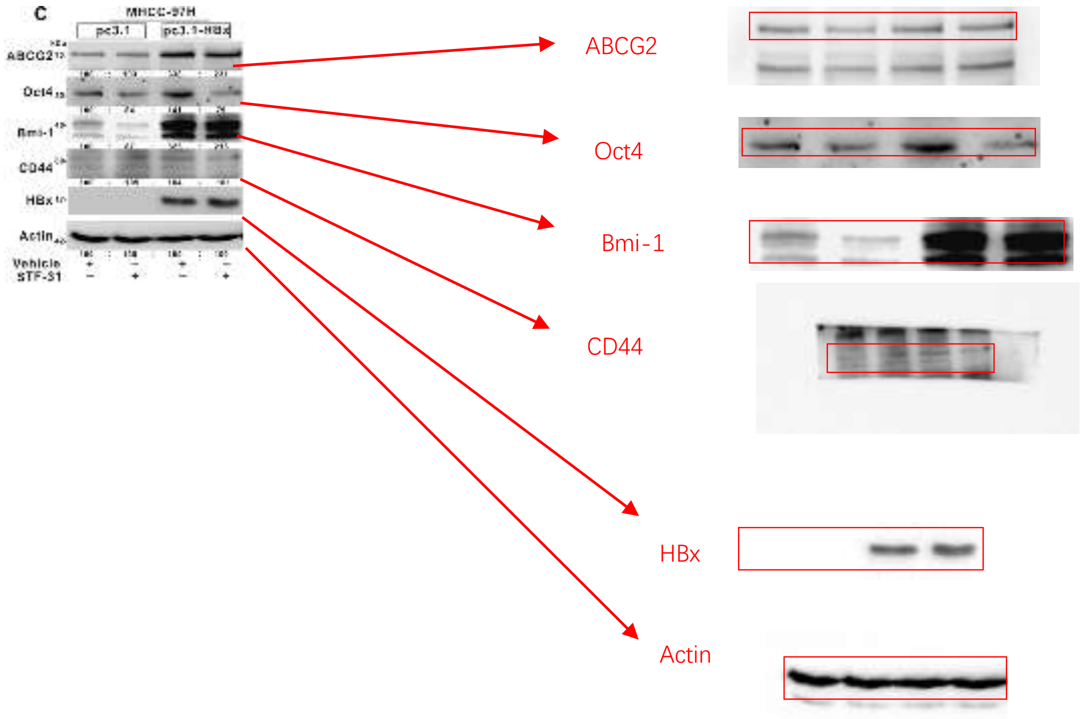
C
MHCC-97H
pc3.1
pc3.1-HBx
ABCG2
Oct4
Bmi-1
CD44
HBx
Actin
Vehicle
STF-31
ABCG2
Oct4
Bmi-1
CD44
HBx
Actin

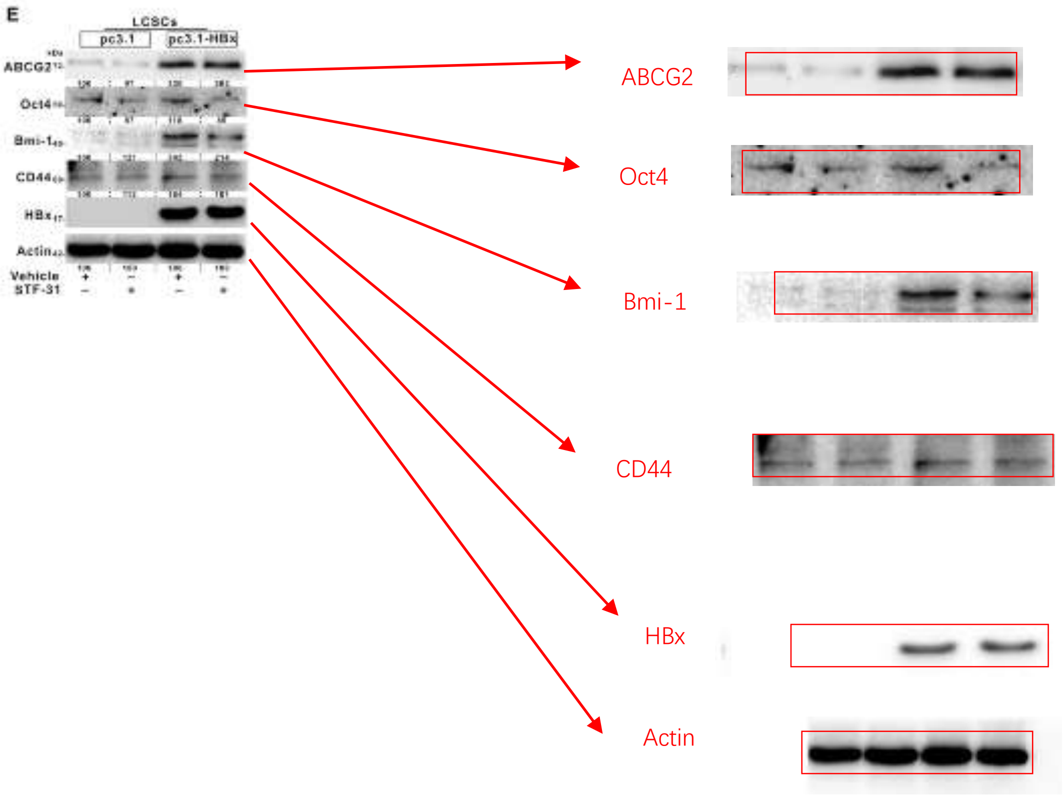
E
LCSCs
pc3.1
pc3.1-HBx
ABCG2
Oct4
Bmi-1
CD44
HBx
Actin
Vehicle
STF-31
ABCG2
Oct4
Bmi-1
CD44
HBx
Actin

F

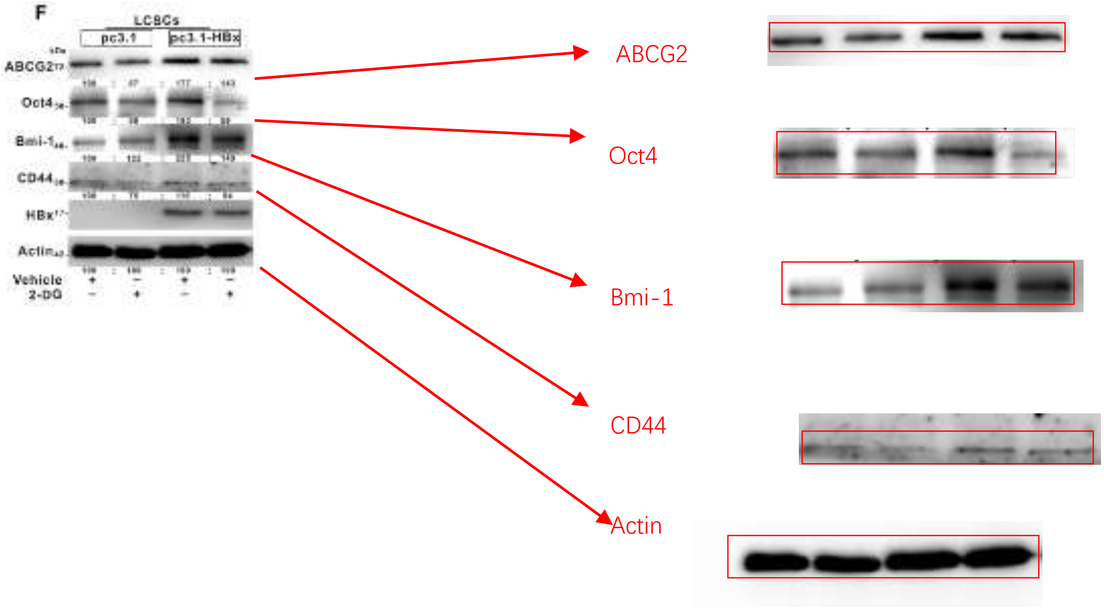
LCSCs
pc3.1
pc3.1-HBx
ABCG2
Oct4
Bmi-1
CD44
HBx
Actin
Vehicle
2-DG
ABCG2
Oct4
Bmi-1
CD44
Actin

Fig 5

C

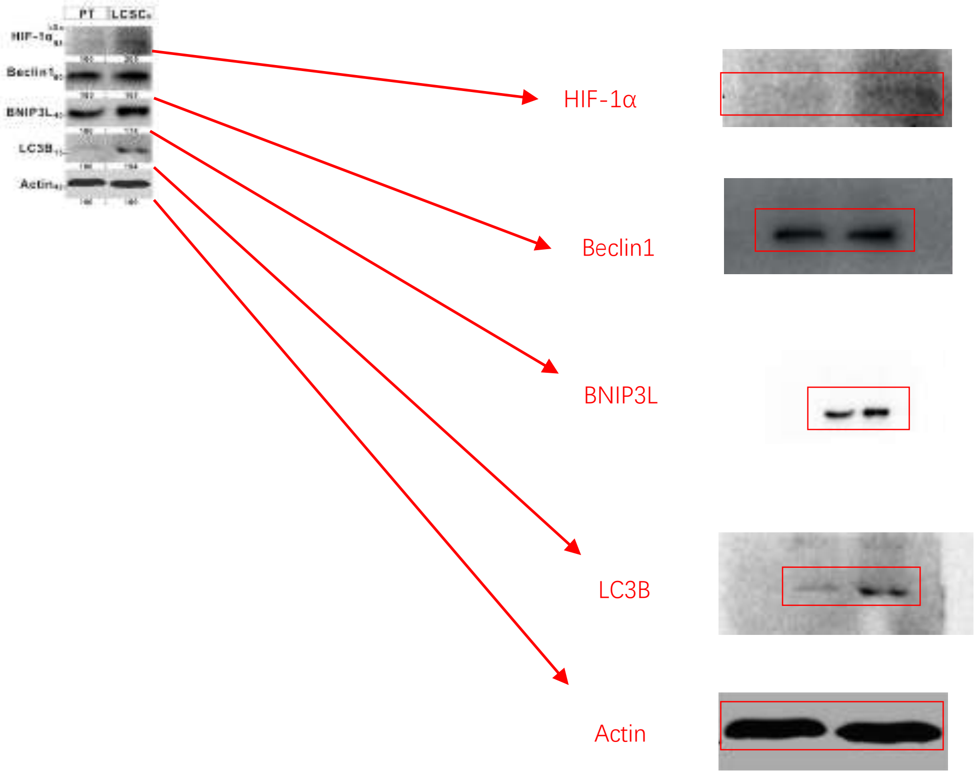
PT
LCSCs
HIF-1α
Beclin1
BNIP3L
LC3B
Actin
HIF-1α
Beclin1
BNIP3L
LC3B
Actin

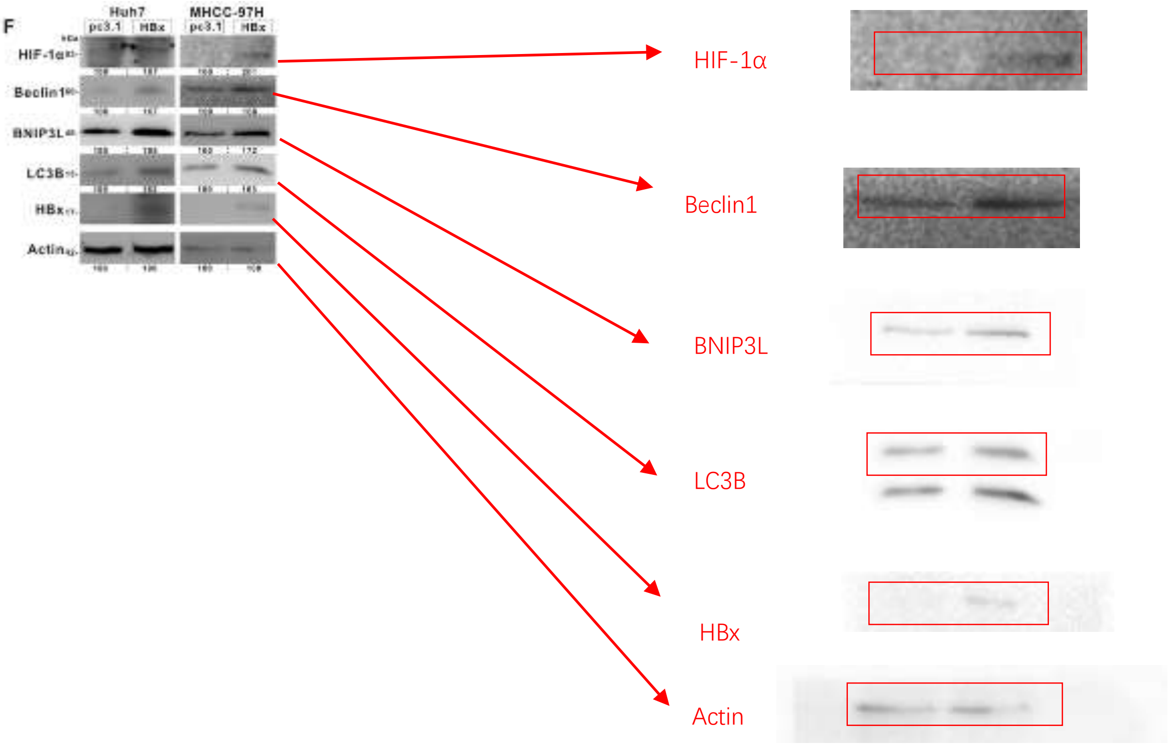
F
Huh7
MHCC-97H
pc3.1
HBx
HIF-1α
Beclin1
BNIP3L
LC3B
HBx
Actin
HIF-1α
Beclin1
BNIP3L
LC3B
HBx
Actin

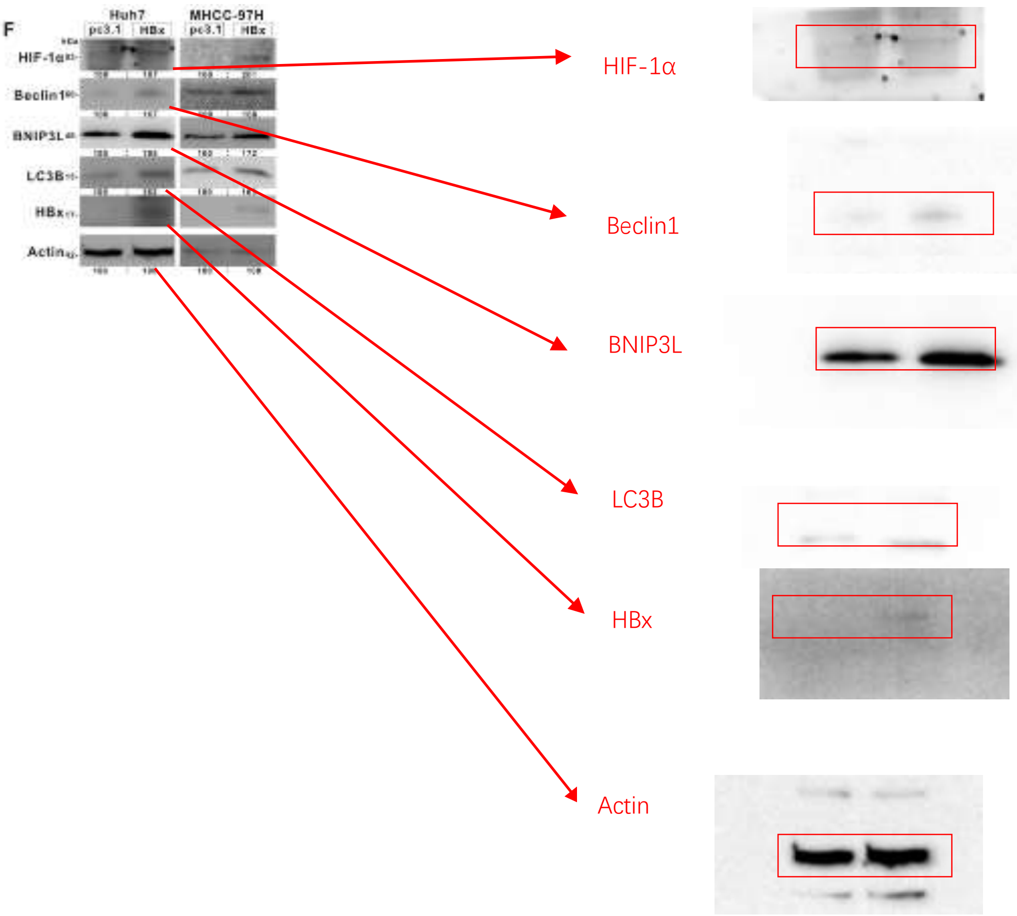
F
Huh7
MHCC-97H
pc3.1
HBx
HIF-1α
Beclin1
BNIP3L
LC3B
HBx
Actin
HIF-1α
Beclin1
BNIP3L
LC3B
HBx
Actin

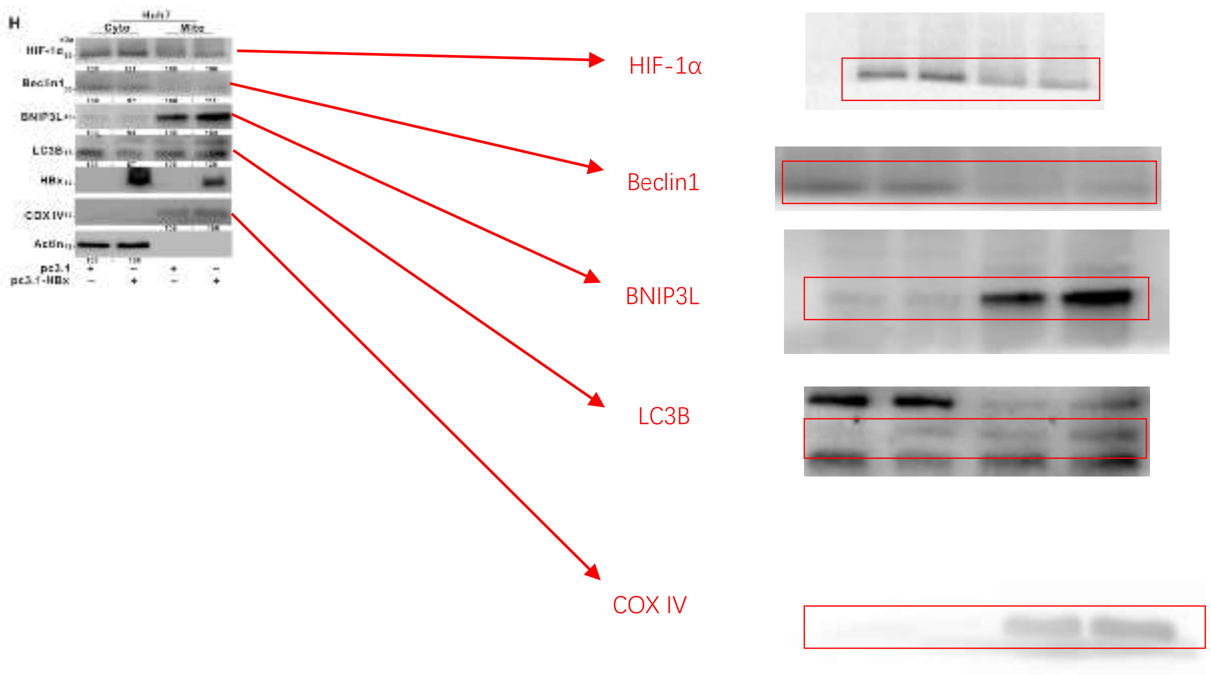
H
HIF-1α
Beclin1
BNIP3L
LC3B
COX IV

Fig 6

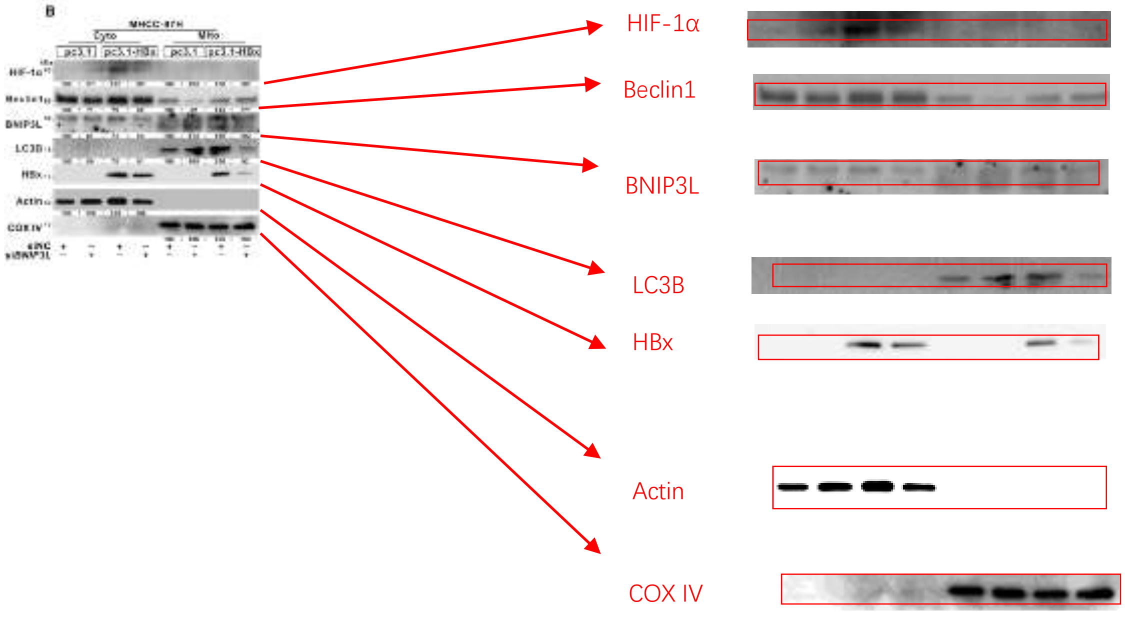
B
HIF-1α
Beclin1
BNIP3L
LC3B
HBx
Actin
COX IV

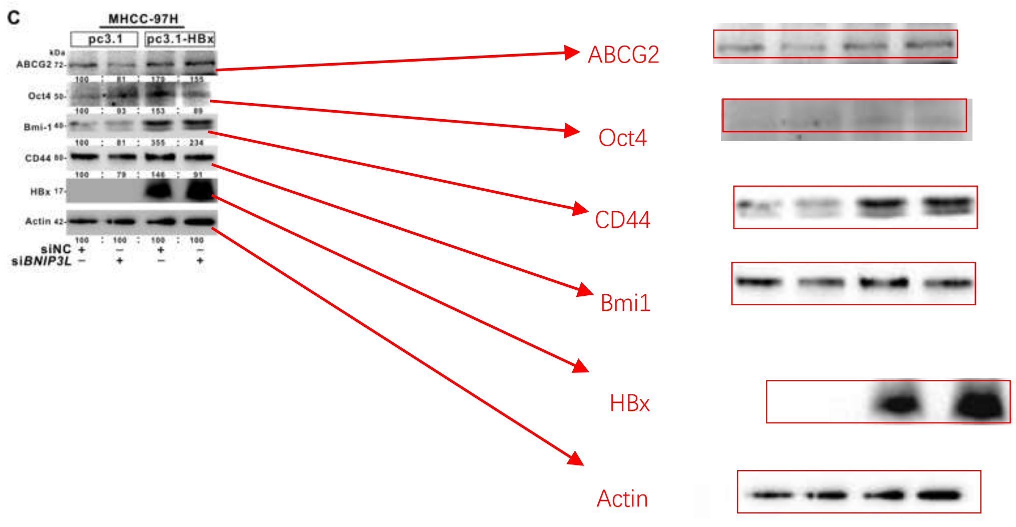
C
MHCC-97H
pc3.1
pc3.1-HBx
ABCG2
Oct4
Bmi-1
CD44
HBx
Actin
siNC
siBNIP3L
ABCG2
Oct4
CD44
Bmi1
HBx
Actin

Fig 8

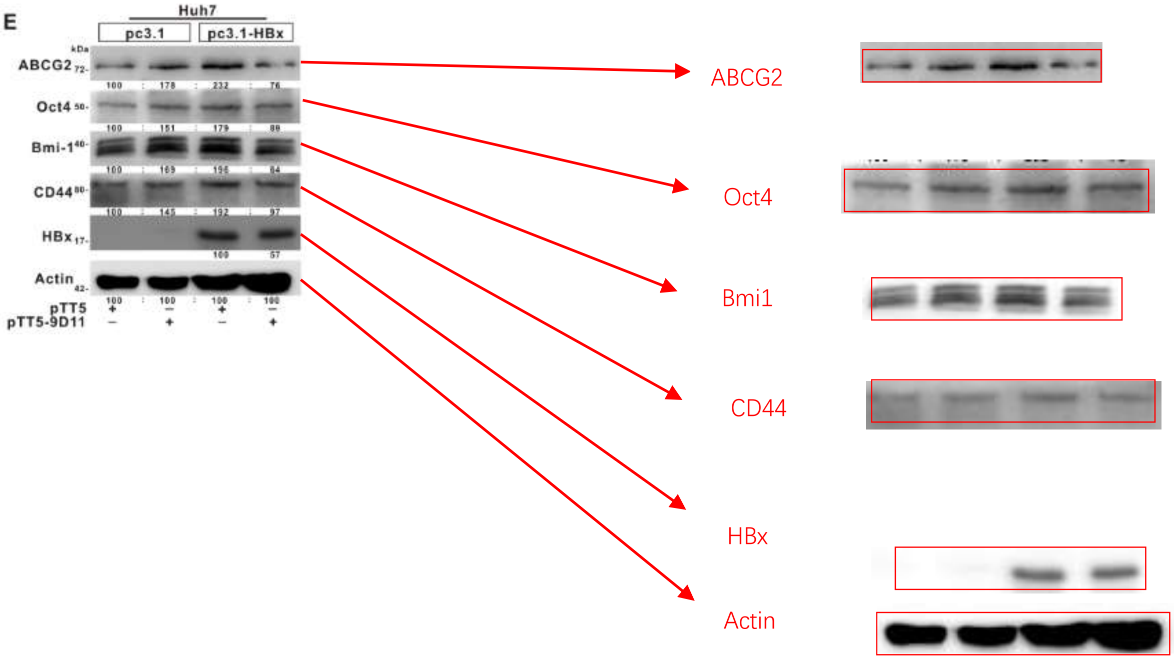
E
Huh7
pc3.1
pc3.1-HBx
ABCG2
Oct4
Bmi-1
CD44
HBx
Actin
pTT5
pTT5-9D11
ABCG2
Oct4
Bmi1
CD44
HBx
Actin

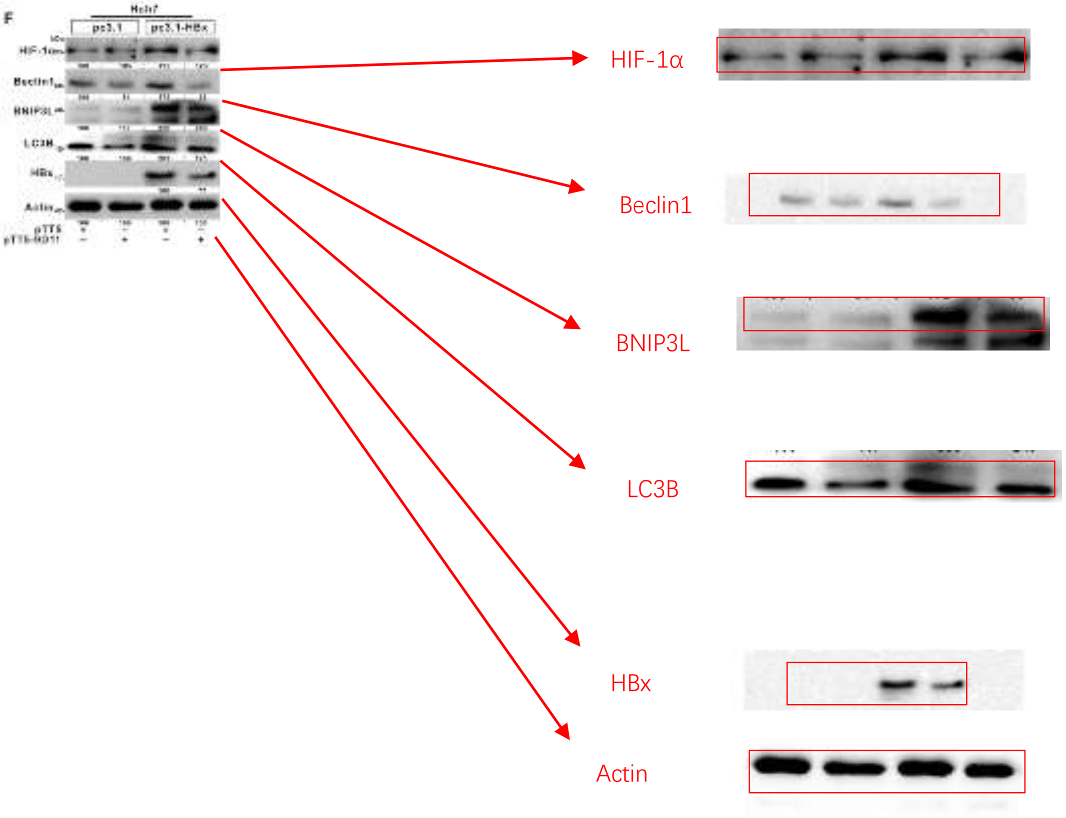
F
HIF-1α
Beclin1
BNIP3L
LC3B
HBx
Actin
